# Structural Biology of Bacterial RNA Polymerase

**DOI:** 10.3390/biom5020848

**Published:** 2015-05-11

**Authors:** Katsuhiko S. Murakami

**Affiliations:** Department of Biochemistry and Molecular Biology, The Center for RNA Molecular Biology, The Pennsylvania State University, University Park, PA 16802, USA; E-Mail: kum14@psu.edu; Tel.: +1-814-865-2758

**Keywords:** bacterial RNA polymerase, transcription, core enzyme, holoenzyme, σ factor, transcription factor, anti-σ factor, X-ray crystallography, NMR, inhibitor antibiotic

## Abstract

Since its discovery and characterization in the early 1960s (Hurwitz, J. The discovery of RNA polymerase. *J. Biol. Chem*. **2005**, *280*, 42477–42485), an enormous amount of biochemical, biophysical and genetic data has been collected on bacterial RNA polymerase (RNAP). In the late 1990s, structural information pertaining to bacterial RNAP has emerged that provided unprecedented insights into the function and mechanism of RNA transcription. In this review, I list all structures related to bacterial RNAP (as determined by X-ray crystallography and NMR methods available from the Protein Data Bank), describe their contributions to bacterial transcription research and discuss the role that small molecules play in inhibiting bacterial RNA transcription.

## 1. Early Research on the Structure of Bacterial RNA Polymerase

**Figure 1:**
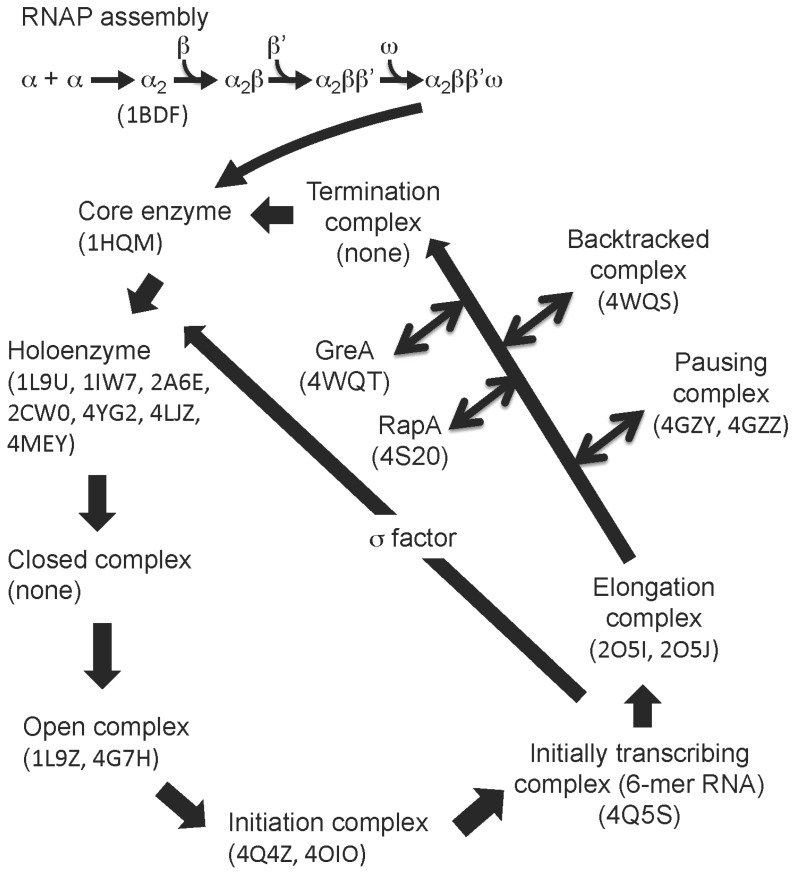
RNAP assembly and transcription cycle. The figure shows the general scheme for bacterial RNAP assembly and the transcription cycle. PDB codes of RNAP structures (in brackets) belonging to each phase of the transcription cycle.

**Figure 2:**
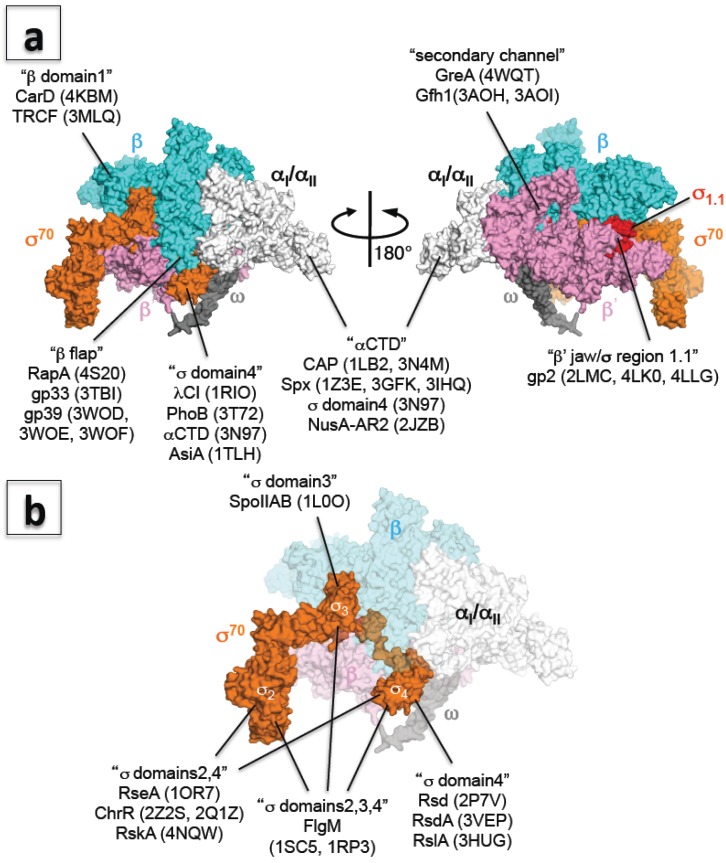
(**a**) Three-dimensional representation of the interaction between RNAP and transcription factors. The *E. coli* RNAP holoenzyme is shown as a molecular surface representation (α subunits: white; β subunit: cyan; β’ subunit: pink; ω subunit: gray; σ^70^: orange; σ region 1.1: red). Transcription factors binding sites are indicated in double quotation marks and PDB codes of structures are shown in brackets; (**b**) Three-dimensional representation of the interaction between σ and anti-σ factors. *E. coli* RNAP holoenzyme is shown as a molecular surface representation, and only the core enzyme is partially transparent (α subunits: white; β subunit: cyan; β’ subunit: pink; ω subunit: gray; σ^70^: orange). Targets of anti-σ factors are indicated in double quotation marks and PDB codes of structures are shown in brackets.

The common core of multi-subunit RNAP in cellular organisms is composed of five subunits that are conserved in all three domains of life. Bacterial RNAP core enzyme is the simplest and best characterized form, consisting of α (two copies), β, β', and ω subunits (Figure 1 and Figure 2a). The core enzyme is responsible for binding to template DNA to synthesize RNA, which is complemented by a σ factor to form a holoenzyme that recognizes the promoter sequence to begin promoter-specific transcription [1,2].

Structural study of bacterial RNAP by electron microscopy [3] began in the mid-1960s. Crystallization of RNAP isolated from *Thermusthermophilus* was first reported in the late 1970s [4]; however, the X-ray crystal structure was not determined until the end of the millennium. Before determining the complete structure of RNAP, stable domains and subcomplexes within RNAP were targeted for structural studies (Table 1). These structures were important guides for building the entire structure of RNAP.

The first atomic view of RNAP was obtained from the C-terminal domain of *Escherichia coli* RNAP α subunit (residues 250–329), also known as αCTD (PDB: 1COO) [5], which plays important roles in regulating transcription via interaction with many transcription factors (Figure 2a) and also binds to the upstream promoter DNA [6]. The structure of αCTD was determined by NMR, which revealed its compact structure and distinct protein topology compared with other DNA binding proteins. The characterization of the structure of αCTD was a springboard for a series of mutagenesis experiments that revealed communication of bacterial RNAP with numerous transcription factors during gene regulation.

**Table 1:** Structural information on bacterial RNAP.

Structure	PDB code	Reference	Source
Subunit and domain			
α subunit NTD	1BDF^X^	[7]	A
α subunit NTD	4NOI^X^	none	F
α subunit CTD	1COO^N^, 3K4G^X^	[5,8]	A
α subunit CTD	1DOQ^N^	[9]	B
α subunit CTD	2MAX^N^	[10]	E
β subunit 2/i4 domains	3LTI^X^	[11]	A
β subunit flap domain	2LY7^N^	none	C
β subunit 1/2 domains	4KBJ^X^	[12]	I
β' subunit i2 domain	2AUJ^X^	[13]	B
β' subunit i6 domain	2AUK^X^, 4IQZ^X^	[13]	A
σ region 1.1	2K6X^N^	[14]	G, a
σ^70^ domain2	1SIG^X^	[15]	A, a
σ^A^ domains2 and 3	1KU2^X^	[16]	B, a
σ^A^ domain4	1KU3^X^	[16]	B, a
σ^A^ domain4–DNA (−35 element)	1KU7^X^	[16]	B, a
σ^A^ domain4	1TTY^N^	[17]	G, a
σ^A^ domain2–DNA (−10 element)	3UGO^X^, 3UGP^X^	[18]	B, a
σ^A^ domain4–αCTD–DNA	3N97^X^	none	B, a, A
σ^N^RpoN–DNA (−24 element)	2O8K^N^, 2O9L^N^	[19]	D, d
σ^N^ core binding domain	2K9M^N^	[20]	D, d
σ^N^RpoN domain	2AHQ^N^	[21]	D, d
σ^E^domain4–DNA (−35 element)	2H27^X^	[22]	A, c
σ^C^ domain2	2O7G^X^	[23]	I, c
σ^C^ domain4	2O8X^X^	[23]	I, c
σ^D^ domain4	3VFZ^X^	[24]	I, c
δ subunit NTD	2KRC^N^, 4NC7^X^, 4NC8^X^, 2M4K^N^, 2KRC^N^	[25,26,27]	C
ε subunit	4NJC^X^	[28]	C
RNAP			
Core enzyme	1HQM^X^	[29,30]	B
Core enzyme (Δω subunit)	2GHO^X^	[31]	B
Holoenzyme	1L9U^X^, 1IW7^X^, 2A6E^X^, 2CW0^X^	[32,33,34]	B
Holoenzyme	4YG2^X^, 4LJZ^X^, 4MEY^X^	[35,36,37]	A
Holoenzyme–DNA (−41 ~ −7)	1L9Z^X^	[38]	B
Holoenzyme–DNA (−12 ~ +12)	4G7H^X^, 4G7O^X^	[39]	B
*de novo* initiation complex	4Q4Z^X^, 4OIO^X^	[40,41]	B
Initially transcribing complex	4Q5S^X^	[40]	B
Elongation complex	2O5I^X^, 2O5J^X^	[42,43]	B
Paused elongation complex	4GZY^X^, 4GZZ^X^	[44]	B
Backtracked elongation complex	4WQS^X^	[45]	B

A: *Escherichia coli*; B: *Thermus aquatics/Thermus thermophilus*; C: *Bacillus subtilis/Bacilus stearothermophilus*; D: *Aquifex aeolicus*; E: *Helicobacter pylori*; F: *Campylobacter jejuni*; G: *Thermotoga maritime*; I: *Mycobacterium tuberculosis*; a: group I σ factor; c: extracytoplasmic function (ECF) σ factor; d: σ^N^/σ^54^ factor; ^X^: X-ray crystallography method; ^N^: NMR method.

A subsequent study revealed the X-ray crystal structure of the α subunit N-terminal domain (αNTD) (PDB: 1BDF) [7]. The structure showed the α subunit homodimer, which is an essential platform for binding of the largest subunits, β and β' (Figure 1).

β and β' subunits form the catalytic center of RNA synthesis and also provide binding sites for double-stranded downstream DNA, DNA/RNA hybrid formed during transcription and RNA. These subunits are highly conserved in bacteria; however, large sequence insertions found in these subunits characterize specific evolutionary lineages of bacteria. These insertions can be isolated as stable domains and crystallized for determining X-ray structures (Table 1). These structures have contributed to providing atomic images of bacterial RNAP because these lineage-specific insertions are located on the peripheral surface of RNAP and electron density maps of these domains are of relatively poor quality in the bacterial RNAP crystals.

σ factor transiently associates with the core enzyme for promoter recognition and it dissociates from the core enzyme once RNAP starts processive RNA synthesis (Figure 1). Proteolysis of σ factor determines its domain organization and structures of some stable domains have been determined by X-ray crystallography and NMR (Table 1). In 1996, the first image of σ factor was obtained from the *E. coli* group I σ^70^ (also known as σ^D^) N-terminal domain containing regions 1.2–2.4 (PDB: 1SIG) [15], which provided insight into the recognition of a −10 element and melting of the promoter DNA by the σ regions 2.4 and 2.3, respectively. A nearly complete view of σ factor was obtained from two proteolytic fragments of *Thermus aquaticus* σ^A^. One fragment contained σ domain 2 (σ2: region 1.2–2.4) and σ domain 3 (σ3: region 3.0–3.1) (PDB: 1KU2), while another fragment contained σ domain 4 (σ4: region 4.1–4.2) (PDB: 1KU3) [16].

## 2. An Explosion of Structural Information on Bacterial RNA Polymerase

The entire structure of bacterial RNAP was first described as a core enzyme form and was isolated from the thermophilic bacterium *T. aquaticus* (PDB: 1HQM) [29]*.* This was an important milestone in the study of bacterial transcription that provided a structural framework for four decades of bacterial transcription research. The structure revealed a unique crab claw-shaped molecule, which was distinct from the T7 phage-like single-subunit RNAP family composed of right-hand-shaped molecules. The configuration of the bacterial RNAP active site was also different from that of the single-subunit RNAP [46], even though these enzymes use the same two-metal ion mechanism [47] for RNA synthesis. Comparison of cellular RNAPs from three domains of life, including eukaryotic RNAPs I [48,49] and II [50] as well as archaeal RNAP [51], revealed a conserved overall shape with multi-subunit arrangement and an active site cleft with conserved motifs including a bridge helix (separating the main and secondary channels), trigger loop (for RNA synthesis and cleavage) and switches (for accommodating DNA and RNA into the RNAP clefts).

## 3. Structural Basis of Transcription Elongation

Crystals of the transcription elongation complex were prepared using *T. thermophilus* RNAP and a synthetic DNA/RNA scaffold, and structures were determined with and without a nucleotide triphosphate substrate (PDB: 2O5I, 2O5J) [42,43]. Structures revealed atomic details of RNAP and DNA/RNA interactions within the DNA binding main channel and the RNA exit channel and showed how the RNAP mobile element trigger loop changes conformation throughout nucleotide substrate selection and phosphodiester bond formation.

During RNA extension, RNAP temporarily stops transcription (transcription pausing) by several mechanisms including σ factor-dependent promoter proximal pausing, elemental pausing, RNA hairpin-dependent pausing and backtrack pausing (Figure 1) [52]. The structure of an elemental-paused elongation complex using *Thermus* RNAP and DNA/RNA scaffolds derived from the *E. coli* his-pause sequence showed a unique RNAP conformation including an open-clamp and kinked bridge helix that may inhibit processive RNA extension (PDB: 4GZY, 4GZZ) [44].

Misincorporated nucleotides in RNA transcripts slow down the rate of transcription and result in the backward movement of RNAP (backtracking), which ejects the 3' RNA end into the secondary channel and later cleaves misincorporated nucleotides by RNAP endonuclease activity (Figure 1). This activity is further stimulated by the elongation factor GreA. The structure of this single-nucleotide-backtracked elongation complex showed a sharply bent RNA backbone at the unpaired RNA base, which accommodated the proofreading cavity of the RNAP active site (PDB: 4WQS) [45]. This study also revealed the structure of RNAP in complex with GreA-like protein, showing that the GreA coiled-coil domain containing acidic residues binds to the RNAP active site through the secondary channel and coordinates Mg^2+^, which is essential for RNA cleavage (PDB: 4WQT).

The transcription factor RapA is an ATP-dependent DNA translocase, which reactivates the stalled elongation complex (Figure 1). The structure of the elongation complex with RapA showed that RapA binds around the RNA exit site, making the RNA channel just wide enough for single-stranded RNA to pass (PDB: 4S20) [53].

## 4. Promoter-Dependent Transcription: How RNA Polymerase Recognizes Promoter DNA Sequences and Initiates Transcription

Bacterial RNAP recognizes promoter DNA and initiates transcription as the holoenzyme form containing the catalytic core enzyme plus the promoter recognition σ factor (Figure 1). Structures of the holoenzyme containing the group I σ factor σ^A^ (also known as SigA), isolated from *T. aquaticus* and *T. thermophilus*, showed that three σ domains (σ domains 2, 3, and 4) arranged on the surface of the core enzyme whose function was to recognize the −35 and−10 elements (separated by ~17 bp DNA) (PDB: 1L9U, 1IW7) [32,33] (Table 1). The structure of the holoenzyme in complex with fork junction promoter DNA (−41 to −7 relative to the transcription start site at +1) showed that the promoter DNA lies across one face of the holoenzyme and is positioned completely outside the RNAP cleft (PDB: 1L9Z) [38]. Universally conserved aromatic residues in the σ region 2.3 are ideally positioned to stack on the exposed face of the base pair at the upstream edge of the transcription bubble.

Atomic details of −35 element recognition by σ domain 4 (σ4), as revealed by co-crystal structure, showed that the helix-turn-helix motif of σ4 reads the hexameric DNA sequence (PDB: 1KU7) [16]. Details of the interaction between σ2 and the −10 element were obtained from the structure of σ2 bound to the single-stranded −10 element (PDB: 3UGO, 3UGP) [18] and the *T. thermophilus* RNAP holoenzyme in complex with the promoter DNA fragment (−12 to +12 on DNA) (PDB: 4G7H, 4G7O) [39]. The holoenzyme/DNA complex structure also showed interactions between σ region 3.2 and template DNA, as well as the presence of core enzyme and non-template DNA in the transcription bubble in order to position the transcription start site of the template DNA at the RNAP active center for initiation of transcription.

Cellular RNAPs start transcription by *de novo* RNA priming (Figure 1). Structures of the *T. themophilus de novo* transcription initiation complex revealed unique contact by the initiating NTPs bound at the transcription start site with template DNA and with RNAP (PDB: 4Q4Z, 4OIO) [40,41]. The RNAP promoter DNA complex was active in the crystalline state and capable of transcribing RNA to 6-mer lengths, allowing to determine the structure of the initial transcription complex (PDB: 4Q5S) [40]. The structure showed that RNAP-RNA contacts stabilized the short RNA transcript in the active site cleft and that the RNA 5' end displaced the σ finger from its position near the active site, which was suggested as a first step in σ release from the RNAP.

## 5. Structures of Alternative σ Factors

Bacterial RNAP uses a group I σ factor (σ^70^ in *E. coli* and σ^A^ in other bacteria) for transcribing housekeeping genes required for log-phase growth. Other classes of σ factors, including the group 2 σ factors (σ^S^, σ^H^, and σ^F^ in *E. coli*), the extracytoplasmic function (ECF) σ factors (σ^E^ and FecI in *E. coli*), and σ^N^ (also known as σ^54^), direct RNAP to genes for induction of stress responses, flagella synthesis, and spore formation in spore-forming bacteria, such as *Bacillus*. Structures of these alternative σ factors were determined as stable domains (Table 1) and provided insights into the mechanism of promoter DNA recognition. Structures of some alternative σ factors have been determined as σ/anti-σ factor complexes, and these structures will be described below (Table 2 and Figure 2b). To date, there has been no high-resolution structure that supports bacterial holoenzyme having any alternative σ factor.

**Table 2:** Structural information on bacterial RNAP and transcription factor complexes.

Structure	PDB	References	Source
σ/anti-σ complex			
σ^F^ domains2, 3, 4/FlgM	1SC5^X^, 1RP3^X^	[54]	D, b
σ^E^ domains2, 4/ChrR	2Z2S^X^, 2Q1Z^X^	[55]	J, c
σ^E^ domains2, 4/RseA	1OR7^X^	[56]	A, c
σ^K^ domains2, 4/RskA	4NQW^X^	[57]	I, c
σ^F^ domain3/SpoIIAB	1L0O^X^	[58]	C, b
σ^70^ domain4/Rsd	2P7V^X^	[59]	A, a
σ^D^ domain4/RsdA	3VEP^X^	[24]	I, c
σ^L^ domain4/RslA	3HUG^X^	[60]	I, c
Transcription factor complex			
αCTD–CAP–DNA	1LB2^X^, 3N4M^X^	[61]	A
αCTD–Spx	1Z3E^X^, 3GFK^X^, 3IHQ^X^	[62,63,64]	C
αCTD–NusA (AR2)	2JZB^N^	none	H, A
β subunit 1/2 domains–CarD	4KBM^X^	[12]	I
β subunit 1 domain–TRCF	3MLQ^X^	[65]	B
σ^A^ domain4–λcI–DNA	1RIO^X^	[66]	B
σ^70^ domain4–PhoB–β flap–DNA	3T72^X^	[67]	A
Holoenzyme–CAP–DNA	3IYD^C^	[68]	A
Core enzyme–GreA/Gfh1	4WQT^X^	[45]	B
Elongation complex–Gfh1	3AOH^X^, 3AOI^X^	[69]	B
Elongation complex –RapA	4S20^X^	[53]	A
Phage factor complex			
Holoenzyme–gp2	4LK0^X^, 4LLG^X^	[36]	A
β' subunit jaw domain–gp2	2LMC^N^	[70]	A
β subunit flap domain–gp33	3TBI^X^	[71]	A
Holoenzyme–gp39	3WOD^X^	[72]	B
β subunit flap domain –gp39	3WOF^X^, 3WOE^X^	[72]	B
σ^70^ domain4–AsiA	1TLH^N^	[17]	A, a
σ^A^ domain4–gp67	4G8X^X^	[73]	K, a

A: *Escherichia coli*; B: *Thermus aquatics/Thermus thermophilus*; C: *Bacillus subtilis/Bacilus stearothermophilus*; D: *Aquifex aeolicus*; H: *Yersinia pseudotuberculosis*; I: *Mycobacterium tuberculosis*; K: *Staphyloccus aureus*; a: group I σ factor; b: group II σ factor; c: extracytoplasmic function (ECF) σ factor; ^X^: X-ray crystallography method; ^C^: Cryo-EM single-particle analysis method; ^N^: NMR method.

## 6. A New Era of Structural Study of Bacterial Transcription Using *E. coli* RNA Polymerase

Crystal structures of bacterial RNAPs have been determined using the *Thermus* genus. Due to a high degree of RNAP sequence conservation among all bacterial species, mechanistic insight derived from the *Thermus* RNAP structure has been generalized to represent the transcription apparatus in all bacteria. Nevertheless, understanding the structure of *E. coli* RNAP is essential to fully interpret the enormous amount of biochemical, biophysical, and genetic data that has been collected on *E. coli* RNAP. In 2013, the first crystal structure of the *E. coli* RNAP σ^70^ holoenzyme was determined (PDB: 4YG2) [35]. *E. coli* RNAP can be readily prepared using an overexpression system, which allows RNAP structural study to move in a new direction using RNAP mutants.

**Figure 3:**
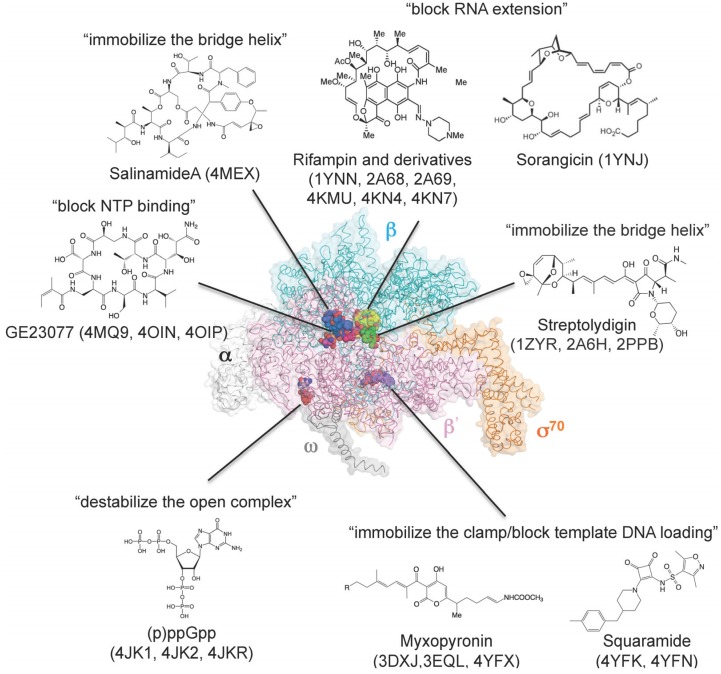
Three-dimensional representation of targets of small-molecule RNAP inhibitors. *E. coli* RNAP holoenzyme is depicted as an α-carbon backbone traced with partially transparent molecular surfaces (α subunits: white; β subunit: cyan; β' subunit: pink; ω subunit: gray; σ^70^: orange). Small-molecule inhibitors bound to RNAP are depicted as CPK models. Chemical structures of small-molecule inhibitors and mechanisms of transcription inhibition are indicated.

Characterization of *E. coli* RNAP has created new possibilities for the structural study of bacterial RNAP. Sixteen structures of *E. coli* RNAP with transcription factors or small molecules have been determined already, including the long-awaited structure of *E. coli* RNAP in complex with (p)ppGpp, a master regulator of stress response in bacteria (PDB: 4JK1, 4JK2, 4JKR) [74,75] (Table 3 and Figure 3). The ppGpp binding site of *T. thermphilus* RNAP was also determined from the crystal structure of the *T. thermphilus* RNAP–ppGpp complex (PDB: 1SMY) [76], but it failed to verify its relevance to *E. coli* transcription regulation by ppGpp [77].

## 7. Transcription Regulation: How RNA Polymerase Communicates with Transcription Factors

Binding of alternative σ factors to the RNAP core enzyme is regulated by anti-σ factor, which forms a stable complex with its target σ factor and blocks core RNAP binding. Structures of the σ/anti-σ factor complex have shown various ways that the core enzyme-binding interface of σ factor can be masked (Table 2 and Figure 2b). The σ/anti-σ complex has also contributed to providing high-resolution images of σ factor, since σ factor is notorious for having a heterogenous conformation and is difficult to crystallize in isolation.

Bacterial RNAP physically interacts with protein factors during gene regulation. Most bacterial transcription factors bind upstream of promoter DNA and recruit RNAP to target promoters and/or facilitate formation of the open complex. Three crystal structures of a transcription factor, RNAP domain, and DNA ternary complex have provided insights into how these factors influence transcription activation through different mechanisms (Table 2 and Figure 2a).

Catabolite activator protein (CAP) (also known as cAMP receptor protein CRP) is the most well characterized bacterial transcription factor, providing insights into the interaction between the helix-turn-helix motif and DNA, the allosteric control of DNA binding, and transcription activation. In classical lactose operon regulation [78], CAP binds DNA ~60 bp upstream from the transcription start site (class I transcription factor binding site) and interacts with αCTD [6,79]. The crystal structure of the CAP–αCTD–DNA ternary complex provided a first view of the transcription factor–RNAP interaction as well as the αCTD–DNA interaction (PDB: 1LB2) [61]. The structure showed that these proteins interact with small interfaces, but it is sufficient for RNAP to target the promoter. A low-resolution view of the transcription initiation complex including *E. coli* RNAP, CAP, and promoter DNA has been determined by cryo-electron microscopy (EMD ID, EMD-5127; PDB: 3IYD) [68].

Bacteriophage λ cI protein (λcI) plays a central role in the lytic to lysogenic growth switch and is able to both activate and repress transcription. For activation of transcription, λcI binds just upstream of the −35 element (class II transcription factor binding site) and interacts with σ domain 4 (σ4). The crystal structure of the λcI–σ4–DNA ternary complex showed that these proteins also use a small interface for recruiting σ4 to the −35 element (PDB:1RIO) [66].

PhoB is a classical two-component response regulator, which interacts with σ4 to activate gene expression. In contrast with λcI-dependent transcription activation, PhoB-dependent promoters lack a canonical −35 element. The structure of the PhoB–σ4–DNA ternary complex showed that contact between σ4 and the DNA major groove is less extensive; however, interaction between σ4 and PhoB compensates for this, allowing recruitment of RNAP to a target promoter lacking the −35 element (PDB: 3T72) [67].

Spx is a global transcription regulator in *Bacillus subtilis* that interacts with a large interface on αCTD, thereby forming a stable Spx–αCTD complex without a DNA platform. Spx regulates gene expression under conditions of disulfide stress, which is sensed by disulfide bond formation between two cysteine residues in Spx. Crystal structures of both oxidized and reduced forms of the Spx–αCTD complex provided insight into how the disulfide bond affects DNA binding (PDB: 1Z3E, 3GFK, 3IHQ) [62,63,64].

Bacteriophages produce transcription factors that bind directly with host RNAPs and hijack host transcription machinery to transcribe phage genomes. Several structural models of bacterial RNAP in complex with phage proteins have been used to explain how phage protein remodels RNAP to redirect transcription machinery to the phage genome (Table 2 and Figure 2a) [17,36,70,71,72,73].

## 8. How Small Molecules Inhibit RNA Transcription

Bacterial RNAP is essential for cell growth and viability, and lack of sequence and structural similarities between certain areas of bacterial and eukaryotic RNAP make this enzyme an attractive target for antibiotic development. Crystal structures of bacterial RNAP in complex with small molecule transcription inhibitors have been used to characterize binding sites, mechanisms of action, and mechanisms of resistance (Table 3 and Figure 3). Currently, two bacterial RNAP inhibitors, rifampin (also known as rifampicin) and fidaxomicin (also known as DIFICID^®^ and lipiarmycin), have been used in clinical practice. Rifampin, a semisynthetic rifamycin, is the cornerstone of current tuberculosis treatment. Structures of the RNAP–rifampin complex (*T. aquaticus* core enzyme, PDB: 1YNN; *E. coli* holoenzyme, PDB: 4KMU) provided a detailed view of the interaction between rifampin and the β subunit and explained how rifampin blocks RNA extension [80,81]. The rifampin binding site is also recognized by sorangicin, which prevents extension of short RNA (PDB: 1YNJ) [82]. Chemical modifications of rifampin have provided an additional RNAP-drug interface that may enhance drug affinity and efficacy, as well as reduce the frequency of spontaneous resistance mutations. Structures of RNAP holoenzymes in complex with rifampin derivatives (*T. thermophiles* holoenzyme, PDB: 2A68, 2A69; *E. coli* holoenzyme, PDB: 4KN4, 4KN7) showed an additional interaction with the σ region 3.2 (σ finger) that may influence binding of template DNA at the active site, thereby reducing the efficiency of transcription initiation [81,83]. The structure of RNAP in complex with GE23077 (PDB: 4MQ9, 4OIN, 4OIP), a drug that inhibits nucleotide binding, provided a framework for creating a bipartite compound by tethering rifampin with GE23077, which has superior affinities for both rifampin-susceptible and rifampin-resistant RNAPs (PDB: 4OIR) [41].

**Table 3:** Structural information on bacterial RNAP and small molecule inhibitor complexes *.

Structure	PDB	Reference	Source
Core enzyme–rifampin	1YNN	[80]	B
Holoenzyme–rifampin	4KMU	[81]	A
Holoenzyme–rifampin derivatives	2A68, 2A69	[83]	B
Holoenzyme–rifampin derivatives	4KN4, 4KN7	[81]	A
Holoenzyme–ppGpp	1SMY	[76]	B
Holoenzyme–ppGpp	4JK1, 4JKR	[74,75]	A
Holoenzyme–pppGpp	4JK2	[74]	A
Core enzyme–sorangicin	1YNJ	[82]	B
Holoenzyme–streptolydigin	1ZYR, 2A6H	[34,84]	B
Elongation complex–streptolydigin	2PPB	[42]	B
Holoenzyme–myxopyronin	3DXJ, 3EQL	[85,86]	B
Holoenzyme–myxopyronin	4YFX	[87]	A
Holoenzyme–GE23077	4MQ9	[41]	B
Holoenzyme–DNA–GE23077	4OIN, 4OIP	[41]	B
Holoenzyme–DNA–GE23077/rifamycin SV	4OIR	[41]	B
Holoenzyme–salinamide A	4MEX	[37]	A
Holoenzyme–squaramide	4YFK, 4YFN	[87]	A

A: *Escherichia coli*; B: *Thermus aquatics/Thermusthermophilus*; * All structures in this table were determined by X-ray crystallography method.

Streptolydigin binds to key elements of the RNAP active site (bridge helix and trigger loop) and inhibits all reactions involved in RNAP transcription by blocking conformational changes in these elements that occur during transcription. Structures of the RNAP–streptolydigin complex revealed that it traps the bridge helix and trigger loop in straight and unfolded conformational states, respectively, by direct interaction with these elements (PDB: 2PPB, 1ZYR, 2A6H) [34,42,84].

Myxopyronin binds to the RNAP switch region, which is a hinge of the RNAP clamp whose function is to open and close the DNA-binding channel. Upon binding, myxopyronin inhibits transcript initiation by preventing formation of the promoter open complex. Structural and biochemical studies on the *T. thermophilus* RNAP–myxopyronin complex (PDB: 3DXJ, 3EQL) have proposed two models of inhibition: myxopyronin inhibits transcription (1) by preventing the opening of the RNAP clamp that permits entry and unwinding of DNA (hinge jamming mechanism) [85] or (2) by interfering with interactions between RNAP and the unwound DNA template strand [86].

A new structure of a bacterial RNAP–inhibitor complex was obtained by using salinamide, which binds the N-terminal end of the bridge helix and prevents nucleotide addition (PDB: 4MEX) [37]. Squaramide is a non-natural bacterial RNAP inhibitor isolated by high-throughput screening, and its binding site was predicted at the switch region, based on analysis of squaramide-resistant RNAP mutants followed by computational modeling using the structure of the *T. thermophilus* RNAP–myxopyronin complex [88]. Crystal structures of *E. coli* RNAP in complex with squaramides were determined, structurally confirming that squaramides bind to RNAP switches (PDB: 4YFK, 4YFN) [87].

During the past decade, our understanding of bacterial transcription processes has drastically improvedas a result of structural studies on bacterial RNAP. I hope this review will be a useful reference for researchers who study the mechanism, structure and function of bacterial RNAP transcription and that structures presented in this review will provide guidelines for designing new experiments.
